# The Cyclopædia of Practical Surgery, &c.

**Published:** 1842-10-01

**Authors:** 


THE CYCLOPÆDIA OF PRACTICAL SURGERY, &c. &c. Edited by *William B. Costello,* M.D. Member of several Learned Societies, National and Foreign. Part XI. London: Sherwood and Co. 1842.

THE subjects treated in the Part of the Cyclopædia before us are Fistula and Fracture—the former by Dr. Costello, the latter by Dr. Macdonnell, of the Richmond Hospital, Dublin. Both articles are very creditable to their authors. That on fracture deserves particular notice, from the interest and value given to details which might almost have been predicted to be stale and uninstructive.

The article is introduced with some general considerations on Fracture which we need not go into. Dr. Macdonnell properly divides the efficient causes of fracture into external and internal. The only efficient internal cause is muscular action. The frequency of fracture of the patella in this way is familiar. But Dr. Macdonnell relates that "spasms, epilepsy, throwing a stone, and sudden effort to regain the equilibrium of the body, are recorded as having induced fractures of the thigh-bone, the tibia, or the humerus. A furious maniac, confined in a chair at Charenton, threw his head violently forwards, and produced separation between the fifth and sixth cervical vertebræ, with fracture, and died in thirty-six hours." Such extreme instances as the above are happily rare, and not familiar, we dare say, to our readers.

External efficient causes act either directly or indirectly in producing fracture. But sometimes an external cause co-operates with an internal one, the latter acting passively. Thus, in the ankle-joint, the external violence not uncommonly acts through the ligaments which, not yielding, tear off the malleoli.

"A very singular case of this nature occurred in the Richmond hospital under the care of Dr. Hutton, three or four years ago. A young man, of about twenty-five years of age, was thrown to the ground with great violence, in wrestling. On rising he found himself unable to walk from pain in the knee. The joint and limb, for some distance above and below, quickly swelled, and became excessively painful." 242.

Fever set in, and the patient died in three weeks with secondary pleuro-pneumonia.

"The joint was found full of pus, with flakes of lymph; the synovial membrane was of a bright scarlet; and a portion of the spine and articulating surfaces of the tibia, nearly as large as a crown-piece, was found torn from the head of the bone, and adhering to the anterior crucial ligament. The preparation is in the Museum of the Richmond hospital." 242.

Dr. Macdonnell estimates the *relative frequency of fractures of different bones.* He gives a table from the Pennsylvania Hospital, according to which the following would seem to be the order of frequency of the more common fractures:—Leg, Upper extremity, Thigh, Clavicle, Ribs, Skull, Jaws, Patella, Foot, Scapula, Fingers, and Spine.

Possibly the number of fractures of the skull may be underrated—and that of fractures of the scapula overrated. For Dr. Macdonnell justly observes that we hear surgeons talk a good deal of fracture of the neck of the scapula, but we see no authentic dissections of it.

There is a table of two hundred and twenty cases of fracture admitted in one year into Guy's Hospital. The Table is as follows:—

| FRACTURES OF THE | Nos. | | Reported, open, or compound. |
|---|---|---|---|
| Femur | | 48 | |
| Tibia and fibula | 36 | | |
| *Tibia* | 13 | | |
| *Fibula* | 13 | | |
| Leg | 4 | 66 | 10 |
| Humerus | | 30 | 2 |
| Fingers | | 18 | 18 |
| Ribs | | 11 | emphysema. 4 |
| Patella | | 8 | |
| Radius | 4 | | |
| Ulna | 1 | | |
| Olecranon | 2 | | |
| Radius and Ulna | 4 | 11 | 1 |
| Clavicle | | 5 | |
| Skull | | 4 | |
| Ossa Nasi | | 3 | 3? |
| Lower Jaw | | 3 | 3? |
| | | 1 | |
| Great Toe 2, Astragalus 1, Spine 1 | | 4 | 3 |
| Of bones, not mentioned, of difficult diagnosis, as about great joints, &c. | | 8 | 1 |
| | | 220 | |

We have some good remarks on the *displacements* of the portions of fractured bones, which are considered under the head of longitudinal, lateral, rotatory, and angular. Then we have remarks on transverse, oblique, longitudinal, and impacted fractures. Dr. Macdonnell shews that the scepticism expressed by some surgeons with regard to longitudinal fractures of long bones is groundless, there being well-authenticated cases of it, as a consequence of gun-shot wounds.

Dr. Macdonnell alludes to a species of fracture common in early life, in which the bone is bent and broken on one side, but not through. There is angular deformity, more or less, without crepitus or mobility of the bone. He proposes the term "flexed or bent bone," to express it.

"Chaussier and others have recorded many cases of fracture occurring during intra-uterine life. They are usually the consequence of some external violence to which the mother has been exposed before the birth of the child, or of force used at the birth; injudiciously, or of necessity, in effecting delivery by art. Chaussier has met, in the same fœtus, with a great number of fractures, which could not be attributed to violence, and to which he has therefore given the name of spontaneous fractures." 247.

Simple and complicated fractures are next touched on. "Compound" fracture is a term which finds no favour in Dr. Macdonnell's eyes. He deliberately excludes it, by reason of its vagueness, and employs in its stead the designation—"open or exposed fracture." So that we know what it means, we suppose that it does not greatly matter what term we employ. Though, critically speaking, there may be objections to the name of compound fracture, still it is so familiar to the English ear that we doubt its being speedily abandoned.

When speaking of the diagnosis of fracture, Dr. Macdonnell very properly reprehends the practice, too frequently resorted to, of pulling a limb about to *satisfy* the surgeon of the existence of a fracture. Every painful examination is more or less injurious, and unless something decided is to be done in case of a fracture being proved, the best plan is to be careful, and gentle, in our manipulations, and trust to the subsidence of inflammatory swelling clearing up the difficulty.

Dr. Macdonnell makes some good observations on the mode of examining and the signs of fractures, more especially on *crepitus*. He adverts more particularly to the possibility of confounding with true crepitus, the not dissimilar sound originating in the synovial sheaths.

"The crepitus which I may style delusive, is caused by the motion of tendons in their sheaths, and of muscles where they move on one another or neighbouring parts by favour of synovial bursæ; when, the lining membrane of the sheaths or bursæ being affected with chronic inflammation, its secretion is so altered that the tendons or muscles no longer glide smoothly, but fret as they move. This crepitus is not of unfrequent occurrence in cases of supposed fracture. It has been compared to the sensation conveyed by pressing arrow-root in powder, or by the fretting of two surfaces of wax drawn lightly along one another. The crepitus of fracture, on the other hand, is grating, harsh, and dry. The synovial crepitus occurs most frequently in the forearm." 251.

All practical surgeons are, of course, familiar with this pseudo-crepitus. But it is not always so easy to distinguish it from true crepitus as might be imagined. This is the case more particularly about the shoulder-joint; for deep-seated crepitus has not that clear, sharp and decisive sound that crepitus near to the surface has. Where any difficulty of this sort exists, all the circumstances of the case must be taken into consideration, and the diagnosis founded on a comparison of them.

Dr. Macdonnell speaks slightingly of the application of the stethoscope to fractures. In the majority of instances, we think, with him, that we can do better without it. But it may be useful for all that. We have seen it so in cases of fractured rib, and the crepitus has been distinguished by the stethoscope when it could not be so by the hand.

We have a good account of the *Reproduction of Bone* illustrated by several woodcuts, and containing what is known upon the subject. Perhaps this abridgement of an abridgement presents the reader with the pith of the matter:—

"Immediately that a fracture takes place, a quantity of blood, greater or less according to the degree of violence inflicted on the soft parts, is effused between the fragments, under the periosteum, and into the cellular tissue immediately surrounding the bone at the fracture; and, in cases of severe injury, extensively into the tissue separating the muscles, or their fasciculi. Hunter supposed the effused blood to perform an important part in the repair of fracture; by coagulating, becoming organized, and forming the basis of callus; The effusion of blood, however, is merely the mechanical consequence of the rupture of the bloodvessels, and is so far from being a necessary element in the process of repair, that those cases recover most favourably and easily in which the effusion of blood is least, because in them the least violence has been done to the soft parts, and the fragments of the bone retain most nearly their natural relations with regard to one another; and further, the first duty nature imposes upon herself is, to bring about the absorption of the effused blood; which, after a time, wastes and assumes the ochre-colour of apoplexies, and then grows pale and disappears. The secretion of fresh organizable lymph,—the result of the inflammation supervening upon the accident,—forms the first step of a long process of repair, which, in a large bone of an adult human subject, extends over a period of from eight to twelve months. A tumour, largest at the seat of the fracture and gradually diminishing in size above and below this point, soon becomes defined. At first, it consists of the organized lymph, which being gradually removed by absorption, the nutritive vessels of the part deposit in its stead a substance resembling fibro-cartilage, for which cartilage, by degrees, is substituted in the same manner. By-and-by, portions of the cartilage being absorbed, particles of bone secreted by the nutrient capillaries take its place, and in process of time the whole tumour becomes osseous, the ossification proceeding from within outwards. Finally, the new bone acquires greater density and strength than the original shaft, insomuch that, in the event of another fracture of the same bone, it will not be broken at the same point as before. The tumour at the fracture, thus different in its constitution at various periods of its age, is technically called the *callus.*" 253.

We observe that a dissection in the Museum of the College of Surgeons of Dublin, hardly bears out Dupuytren's views of the distinctness between the "provisional" and the "permanent callus." We are inclined to believe, from experiments and dissections of our own, that this distinction is pushed too far, and that it cannot always be verified by an appeal to nature. It is not a very uncommon circumstance, if we may trust our own observations, for the medullary cavity at the seat of injury to be very imperfectly restored.

Most persons are aware, that in a perfectly consolidated fracture the callus is ultimately stronger than the original bone. The following analyses appear to determine the matter. One analysis was made by Dr. Davy, one hundred and twenty-eight days after the injury. The shaft and the callus are compared.

| | Shaft. | Callus. |
|---|---|---|
| Animal matter .. .. .. .. .. | 38·60 | 38·8 |
| Phosphate lime, &c. .. .. .. .. | 61·40 | 61·2 |
| | 100·00 | 100·0 |

Another analysis, at a later period, (not specified,) is quoted from M. Henry Gauthier de Claubry. It shows the final supersaturation of the callus with calcareous matter.

| | Original Bone. | Callus. |
|---|---|---|
| Animal matter .. .. .. .. .. | 56·284 | 43·795 |
| Carbonate of lime .. .. .. .. | 3·846 | 9·785 |
| Phosphate of lime .. .. .. .. | 38·075 | 44·894 |
| Phosphate of Magnesia .. .. .. | 1·012 | 1·526 |

This decadence of the animal matter in the new bone, as it reaches to its permanent state, is analogous with what we see in other tissues. Cicatrices of skin, &c. are well known to have a lower degree of vitality than the original parts.

An Appendix to the account of the Reparation of Bone is furnished by Mr. Wilkinson King. It is to be regretted that this gentleman does not write in a more concise and simple style. His views would be quite as striking and more intelligible. So far as we can see they amount to this —that bone is most easily and naturally formed upon bone, a postulate which, we fancy, will be easily granted—that the periosteum being excited, if you will, inflamed, is the principal agent in the process of deposition, which cannot well be denied—that the quantity of callus is in some degree proportionate to the amount of irritation, and is, therefore greater in an animal who hops about on three legs and disturbs his broken one, than in man who is put to bed with splints upon him—that the solid plug described as descending into the medullary cavity of bones is more talked about than seen, a sentiment with which we agree—that spicula of bone in the midst of callus are in the same problematical category.* After a good many observations, Mr. King sums up:—

---

* The following passage will exhibit both the view and the style of Mr. King:—

"Every museum contains the alleged proofs of these facts; but I may safely ask, where is the collection, in which we may find a series of specimens to establish the spontaneous and independent commencement of bony callus,—the successive stages of irregular or accidental callus? The formation of all bones, by initiative points of ossification, follows peculiar laws; and it is not impossible that the same laws are to be seen in operation in other instances; but the ordinary rule of repairing fracture is parallel to the growth and not to the commencement of bone; it is *bone upon bone,* wherever increased nutrition or inflammation is excited."

"For a brief review of this appended chapter I may repeat, 1st, That the periosteum, whether original, or derived from granulation, adhesion, or cicatrization, is the chief source of the reparation, as of the nutrition of bone, and that the original membrane is only in degree more efficient than the adventitious: 2ndly, That, whatever nidus may precede, the ossification goes on beneath periosteum—bone upon bone—till the fragments are cemented by the union of two portions of new bone: 3rdly, The causes of ossification are inflammation, the presence of bone and the existence of the necessary material in the blood: 4thly, Remodelling absorption finally comes into operation, and its cause is that of the declining nutrition, disease, &c." 273.

Dr. Macdonnell passes to the *Treatment of Fractures*. His remarks and directions with reference to the transport of a person with a broken limb to his home or to the spot where the fracture can be properly reduced and put up, are deserving of every young surgeon's study. We shall give a sketch of them.

Unfortunately, in most instances, the patient is carried by ignorant persons, and the limb is rudely jolted, perhaps materially injured. But, suppose that a stage-coach passenger, or a gentleman, while hunting, has met with a fracture of the thigh. The fracture being ascertained, the surgeon, with three or four assistants, cuts off the boot and rips up the trousers in the outer seam. He then commits the sound limb to one assistant, the other leg to another, while, himself grasping the thigh above and below the fracture, steadies it during the transport. If a third assistant is strong, and the patient not heavy, the trunk may be given to his sole charge. "Let him direct this assistant, turning his face towards the feet of the patient, and having his right arm next him, to take hold of the patient's chest immediately below the axillæ, if possible clasping his hands under the spine, while the patient, with or without the aid of a handkerchief passed over the opposite shoulder of the assistant, helps to support his own body by clinging round the neck of the assistant. I think it of some importance to make these arrangements with a view to carry the patient feet foremost, that he may see the way he is going. He will thus, in the state of nervous alarm in which he is, be less liable to involuntary muscular efforts, which are always productive of mischief, as, not being anticipated by the surgeon, he cannot be prepared to counteract them." The patient now, directed to be perfectly passive, is carried to the nearest house and placed upon a bed.

The surgeon has now to provide temporary substitutes for splints and rollers, and Dr. Macdonnell's are particularly ingenious.

"Let him provide himself with ten or twelve willow, hazel, or other straight rods, about as thick as the little finger, and long enough to run from the great trochanter to the foot. Let each of these be bedded neatly in a small quantity of unbroken straw, and bound with twine. Three of these bound to one another side by side, in three or four places, will form an extempore splint of the length of the limb, and capable of adapting itself to its form. Three or four splints of this kind may be constructed. Cushions for these are next to be made of old linen, or any other soft materials the surgeon can lay his hands on. If tow is to be had, it will be of great use in filling up vacant spaces; if not, any soft material that can be got, must be substituted. A many-tailed bandage is now to be placed on the splint intended for the back of the limb, and a long single-headed roller, or tape, or cord, is to be prepared, to fix the splints when applied.

Dr. Macdonnell selects, we think very properly, the straight position. It must be evident that it is much more secure, and convenient in all respects, than the flexed.

"Next, while the surgeon preserves the reduction with his own hands, he and one assistant raise the whole limb, so as to permit a second to slip the splint, covered with its cushion and the many-tailed bandage, fairly underneath it. The limb is then to be let gently down on the splint, an assistant supporting it, while the surgeon applies the pieces of the many-tailed bandage under the splint; five or six strong tapes or cords should be laid across at regular intervals, for the purpose of binding the splints on the limb when these have been placed in their proper situations. Let a second splint be laid on the inside of the limb, protected by the substitute for a cushion, and a third along the outer and anterior part in the same manner. Perhaps a fourth may be required along the front, when the limb is large. The outer splint should extend from the top of the great trochanter to the point of the external malleolus—the inner from near the fold between the thigh and perineum to the point of the internal malleolus—none of them should descend so low as the sole of the foot, lest they should be liable to catch upon any thing, and by so doing rudely shake the limb. Before binding the strings round the splints, the vacancies between them and the limb, as at the ham, &c. should be filled up, and the prominences of the bones should be protected from undue pressure with tow or any other soft material. Let the tapes be tied firmly, three for the thigh, and two for the leg. Lastly, the single-headed roller is to be applied over all, with a moderate degree of tightness, from the toes to the top of the limb." 275.

The patient is now in a state to be conveyed with tolerable safety, home. He may be transported in a coach, with the limb on the two seats, or on an Irish jaunting-car, &c. But, if the fracture is a very bad one, a litter must be manufactured and hand carriage is requisite. Dr. Macdonnell's recipe for a litter *pro re natâ* is as good as his other prescriptions in this way.

"A door, or any similarly shaped board will answer, with two poles securely nailed across and underneath it, one a little below the top and the other a little above the bottom of the door; or the poles may, in the same way, be secured along each side of the board. In this way two men can carry the litter like a sedan chair, whereas four would be necessary according to the former plan. In either case the poles should extend two or three feet beyond the board, that it may not strike against those who carry it. If nothing of this kind is to be had, a litter may be constructed in the following manner. Let four poles, two, seven or eight, and two, ten or twelve feet long, be procured, if not in the house, from the next wood. Let the upper and lower edges of two strong sheets be sewed well together with pack-thread, and let the two longer poles be then passed through them, and separated, till the sheets, the side of one of which is to be in contact with that of the other, are tensely stretched by the poles. Let them be fixed in that position, by passing the shorter poles under them, immediately above and below the sheets, and nailing or tying them all firmly together at the four points where they cross each other. Lastly, cutting one or other pair of poles near to the sheets, we have a litter requiring two or four men to carry it, according as we cut short the poles. Either may be necessary, according to the weight of the patient. The two-porter litter is always to be preferred where there is a choice. It can be carried through doorways, and the smaller the number of persons employed at the same time in the transport, the less is the risk of want of concert in their movements. A thin hair mattress, or one or two blankets, and a bolster, being laid on a litter, it is now fit to receive the patient." 275.

The surgeon should superintend the removal of the patient—the bearers should keep step like chairmen—and a couple of pillows hollowed in the middle may be placed across under the limb, to give it lateral support.

With regard to the bed, a narrow one with a low foot-board is the best, though we must take what we can get—and a hair mattress is the proper thing to lie upon.

The observations on the reduction and coaptation of a fracture are judicious. Dr. Macdonnell properly reprobates violence or excessive force in the extension of the limb. When there is much muscular resistance it is better to wait for the effects of time and soothing measures, but, in the great majority of cases, the sooner reduction is effected the better. It is very absurd to wait for two or three days, as some people do, when the tonic contraction of the muscles and the coagulation of the blood and lymph have helped to fix the broken portions in a contracted and improper position. More pain and more force in the reduction are the results.

We have full details respecting splints, bandages, cushions and so forth. We observe that Dr. Macdonnell speaks of paste-board splints in the management of fractures in children, but does not seem to be aware that undressed leather has been applied to the same purpose, and is in many respects far preferable.

On one point Dr. Macdonnell insists, and he cannot insist too strongly—not applying the bandages too tightly. Simple as this rule may appear, how often is it infringed in practice! The temptation to put the fracture up *securely* is very great, and great mischief as well as little is constantly inflicted. Dr. Macdonnell relates a striking case, in which a poor boy separated the inferior epiphysis of the radius from its shaft. A medical man put up the limb so tightly as to occasion a good deal of pain. The splints were not loosened and gangrene of the limb supervened. The lad was in imminent danger for some days, but ultimately a line of separation formed a little below the elbow-joint, the bones were sawed through, and the patient recovered. We fear that instances might be found to match this, or worse.

Dr. Macdonnell enumerates the ordinary methods of treating fractures, by extension of the limb in the straight position—by the bent position,—by the starched bandage or pasteboard splints—and by suspension of the limb. He remarks, properly enough, that no one can be employed to the absolute exclusion of the rest, but he prefers the bent position. We must confess that in fractures of the femur in its lower two thirds, and in fractures of the leg, but more particularly in the former, we like the straight position best.

The starched bandage Dr. Macdonnell thinks (and we are of the same opinion) will not maintain its hold upon the Continent: it has never got one here. He himself has known several instances in which much mischief—high inflammation, abscesses, gangrene ensued from its, perhaps injudicious and improper, employment.

Here, we believe that we must stop. The article is incomplete in the Part of the Cyclopædia before us, and remains to be taken up in the next. When that appears we shall resume our notice of what is really a valuable contribution to surgery.

